# The impact of hepatocyte-specific deletion of hypoxia-inducible factors on the development of polymicrobial sepsis with focus on GR and PPARα function

**DOI:** 10.3389/fimmu.2023.1124011

**Published:** 2023-03-16

**Authors:** Tineke Vanderhaeghen, Steven Timmermans, Melanie Eggermont, Deepika Watts, Jolien Vandewalle, Charlotte Wallaeys, Louise Nuyttens, Joyca De Temmerman, Tino Hochepied, Sylviane Dewaele, Joke Vanden Berghe, Niek Sanders, Ben Wielockx, Rudi Beyaert, Claude Libert

**Affiliations:** ^1^ Flanders Institute for Biotechnology (VIB) Center for Inflammation Research, Ghent, Belgium; ^2^ Department of Biomedical Molecular Biology, Ghent University, Ghent, Belgium; ^3^ Department of Clinical Pathobiochemistry, Institute for Clinical Chemistry and Laboratory Medicine, Technische Universität Dresden, Dresden, Germany; ^4^ Deutsche Forschungsgemeinschaft (DFG) Research Centre and Cluster of Excellence for Regenerative Therapies Dresden, Technische Universität Dresden, Dresden, Germany; ^5^ Department of Nutrition, Genetics, and Ethology, Faculty of Veterinary Medicine, Ghent University, Ghent, Belgium; ^6^ Department of Pathology, Bacteriology, and Avian Diseases, Faculty of Veterinary Medicine, Ghent University, Ghent, Belgium

**Keywords:** sepsis, hypoxia, detection, metabolism, glucocorticoids (GCs), PPARalpha

## Abstract

**Introduction:**

Polymicrobial sepsis causes acute anorexia (loss of appetite), leading to lipolysis in white adipose tissue and proteolysis in muscle, and thus release of free fatty acids (FFAs), glycerol and gluconeogenic amino acids. Since hepatic peroxisome proliferator-activated receptor alpha (PPARα) and glucocorticoid receptor (GR) quickly lose function in sepsis, these metabolites accumulate (causing toxicity) and fail to yield energy-rich molecules such as ketone bodies (KBs) and glucose. The mechanism of PPARα and GR dysfunction is not known.

**Methods & results:**

We investigated the hypothesis that hypoxia and/or activation of hypoxia inducible factors (HIFs) might play a role in these issues with PPARα and GR. After cecal ligation and puncture (CLP) in mice, leading to lethal polymicrobial sepsis, bulk liver RNA sequencing illustrated the induction of the genes encoding HIF1α and HIF2α, and an enrichment of HIF-dependent gene signatures. Therefore, we generated hepatocyte-specific knock-out mice for HIF1α, HIF2α or both, and a new HRE-luciferase reporter mouse line. After CLP, these HRE-luciferase reporter mice show signals in several tissues, including the liver. Hydrodynamic injection of an HRE-luciferase reporter plasmid also led to (liver-specific) signals in hypoxia and CLP. Despite these encouraging data, however, hepatocyte-specific HIF1α and/or HIF2α knock-out mice suggest that survival after CLP was not dependent on the hepatocyte-specific presence of HIF proteins, which was supported by measuring blood levels of glucose, FFAs, and KBs. The HIF proteins were also irrelevant in the CLP-induced glucocorticoid resistance, but we found indications that the absence of HIF1α in hepatocytes causes less inactivation of PPARα transcriptional function.

**Conclusion:**

We conclude that HIF1α and HIF2α are activated in hepatocytes in sepsis, but their contribution to the mechanisms leading to lethality are minimal.

## 1 Introduction

Sepsis is defined as a life-threatening organ dysfunction caused by a dysregulated host response to an infection. Despite intensive research, increased awareness and medical improvement, sepsis and septic shock remain an important cause of morbidity and mortality in the intensive care units (ICUs) worldwide (1, 2). The annual global incidence of sepsis is 48,9 million cases with 11 million sepsis-related deaths (3). The current management of sepsis is supportive rather than curative and focusses on controlling the infection, fluid resuscitation, and vasopressor treatment and mechanical support of failing organs (4). Although a lot of clinical trials with immunomodulatory therapies have been performed, none of these therapies have demonstrated survival benefit. The lack of successful, innovative therapeutics might be attributed to the fact that not only a dysregulated inflammatory response, but other pathways, such as metabolic alterations, might also play an important role (5, 6).

Sepsis pathogenesis is characterized by inflammation, immune activation, the acute-phase response, fever, tachycardia and tachypnea, complement activation, and coagulopathy, all of which require a supraphysiological amount of energy (7). Regardless of their increased energy needs, sepsis patients are often unable or unwilling to eat leading to a negative energy balance. Therefore, it is suggested that a starvation response (SR) is induced in sepsis patients (8). When a SR is initiated, carbohydrate and fat reserves are broken down in the liver and muscle, and white adipose tissue (WAT), respectively, to generate ATP and release high-energy metabolites e.g. lactate, free fatty acids (FFAs) and ketone bodies (KBs) (9). These processes are mainly controlled by two transcription factors, namely the glucocorticoid receptor (GR) and the peroxisome proliferator-activated receptor alpha (PPARα) on a transcriptional level (7). However, GR and PPARα become dysfunctional during sepsis, and so the amounts of glycogen, WAT, and muscle mass rapidly decline, while blood levels of FFAs, glycerol, amino acids (AAs), and lactate increase (10–12). This correlates with disease severity and lethality in sepsis patients and animals (10, 11, 13, 14), and learns us that the SR in sepsis might be failing.

On the one hand, a fast and progressive failure of GR functioning leading to GC resistance (GCR) in the liver and in other organs during sepsis contributes to the failing SR. This GCR is strongly associated with a reduced GR DNA-binding capacity and causes a dysfunctional gluconeogenesis in hepatocytes, which leads to hypoglycemia and lactate accumulation in the blood. High lactate levels are not toxic by themselves, but are highly lethal when GCR is present (10). We have also demonstrated that TNF-mediated GCR can be a result of the sequestration of co-factor p300 to NF-κB, thereby preventing its accessibility to GR (15).

Sepsis is also characterized by a PPARα dysfunction in the liver. This dysfunction can, in part, be explained by a rapid decline of hepatic PPARα mRNA and protein levels, which lead to a reduced expression of its target genes involved in FFA β-oxidation and ketogenesis (11, 16). As a consequence of PPARα malfunctioning, ectopic deposition of lipids in the liver and kidney occur during sepsis and thereby cause lipotoxicity and tissue damage rather than production of energy (11).

Besides GR and PPARα dysfunction, sepsis is also characterized by fundamental shifts in tissue metabolism in combination with a decreased tissue perfusion and edema. This might result in decreased oxygen delivery to cells and tissue hypoxia during sepsis (17, 18). The master regulators involved in oxygen homeostasis are hypoxia-inducible factors (HIFs). HIFs are heterodimeric transcription factors consisting of an α- and β-subunit. Three α-subunits are known, namely HIF1α, HIF2α, and HIF3α, of which its expression is known to be oxygen-sensitive, while the β-subunit is constitutively expressed. Under normal oxygen levels, HIFα subunits are hydroxylated by prolyl-4-hydroxylases (PHDs) leading to the binding of the von Hippel-Lindau protein (pVHL) and 26S proteasome degradation. Under hypoxic conditions, or in the absence of its co-factors Fe^2+^, α-ketoglutarate (α-KG) or vitamins, PHDs are inactivated and HIFα hydroxylation is inhibited (19). Besides reduced oxygen availability, inflammation also inhibits PHD activity and will promote the transcription of HIF1α mRNA and HIF activity (20). Once HIF proteins are stabilized, they will regulate the expression of genes involved in glucose metabolism (21), lipid metabolism (22, 23), and erythropoiesis (24). Furthermore, a clear crosstalk between the GR and HIFs exists (25, 26), and hypoxia is associated with increased lipolysis, increased FFA levels in the blood, and affects fatty acid β-oxidation (22, 23, 26).

We hypothesize that cecal ligation and puncture (CLP)-induced polymicrobial sepsis leads to a rapid metabolically changed physiology, leading to an increase in metabolites with high tropism for hepatocytes (β-oxidation and gluconeogenesis) such as FFAs, glycerol, gluconeogenic AAs and lactate. Since HIFs interfere with the expression of multiple important metabolic enzymes, and since HIFs use transcriptional co-factors, such as p300, which are also essential for the function of GR and PPARα, we aimed to investigate the role of HIF1α and HIF2α in more detail during sepsis, in the liver. We have studied HIF activity in the liver of septic mice using a newly generated HIF-luciferase reporter mouse in combination with bulk liver RNA sequencing (RNA-SEQ) data. Furthermore, we have investigated the functional role of HIF1α and/or HIF2α during sepsis in more detail *via* hepatocyte-specific HIF1α and/or HIF2α knock-out mice with a focus on their role in the annihilation of the transcriptional function of GR and PPARα.

## 2 Materials and methods

### 2.1 Mice

Male C57BL/6J mice were purchased from Janvier (Le Genest-St. Isle, France). *HIF1a^fl/fl^*, *HIF2a^fl/fl^* (provided by Prof. Dr. Ben Wielockx) were crossed with *Albumin Cre* transgenic mice, and the offspring was intercrossed to generate *HIF1a^fl/fl^ Albumin Cre^Tg/+^* (HIF1a^AlbKO^), *HIF2a^fl/fl^ Albumin Cre^Tg/+^* (HIF2a^AlbKO^), and *HIF1aHIF2a^fl/fl^ Albumin Cre^Tg/+^* (HIF1aHIF2a^AlbKO^) mice, all in a C57BL/6J background. All offspring was genotyped by PCR on genomic DNA isolated from toe biopsies. Mice were housed in a temperature-controlled, specific pathogen free (SPF) air-conditioned animal house with 14 and 10h light/dark cycles and received food and water *ad libitum.* All mice were used at the age of 8 – 12 weeks, and all experiments were approved by the institutional ethics committee for animal welfare of the Faculty of Sciences, Ghent University, Belgium.

### 2.2 Plasmid and transgene construction

A hypoxia reporter plasmid was purchased from Addgene (plasmid #26731). The plasmid contained a cassette containing three Hypoxia Responsive Elements (HRE) derived from the mouse *Pgk1* gene (sequence HRE: TGTCACGTCCTGCACGACTCTAGT), followed by a mini TK promoter (27), firefly luciferase cDNA and SV40 polyA, flanked by 2 chicken beta-globin HS4 insulator core sequences (28) on both sides. The reporter plasmid is considered to be specific for hypoxia signals (27). The insulators were flanked with 800 bp homology arms to the TIGRE locus (29) and by NotI restriction sites. The 5629 bp cassette was made synthetically (Genscript) and cloned in a pUC57 backbone vector. The cassette was removed from the vector by NotI digest, gel extracted and purified using phenol-chloroform extraction and ethanol precipitation. The fragment was dissolved in TE buffer pH 7.5.

### 2.3 Generation of transgenic mice

The purified fragment (1.5 ng/µl) was injected in C57BL/6J zygotes together with Cas9 protein (60 ng/µl, VIB Protein Core) and cr/tracrRNA duplex to the TIGRE locus (5’ TAACTTTAATTCTAGCGATC 3’, 40 ng/µl). Founders were identified by PCR amplification of toe DNA with primers to the luciferase cDNA identifying integration of the cassette in the genome: 5’ GGAAGACGCCAAAAACATAA 3’ and 5’ GGAAGACGCCAAAAACATAA 3’. Correct integration in the TIGRE locus was identified with a PCR over the left homology region with a primer in the TIGRE locus 5’ GCCTGGAACTCACTATACAA 3’ and a primer in the cassette 5’ TTAATATGCGAAGTGGACCT 3’ on the one hand and a PCR over the right homology region with a primer in the cassette 5’ TAAAAAACCTCCCACACCTC 3’ and a primer in the TIGRE locus 5’ AACTAAGAAGAAACGCCTCC 3’.

### 2.4 Cecal ligation and puncture

Polymicrobial sepsis was induced in mice by performing a CLP procedure, as previously described by Rittirsch et al. (2009) (30). Briefly, mice were anesthetized by isoflurane inhalation and a midline incision was made in the abdomen. Then, the cecum was exposed, 75% ligated, and a single through-and-through puncture was made with a 21-Gauge needle. During the procedure, a small amount of cecal content was extruded. The abdominal musculature and skin were closed by applying simple running sutures and metallic clips, respectively. During lethality experiments, mice were injected intraperitoneally (i.p.) with broad-spectrum antibiotics (25 mg/kg ceftriaxone and 12.5 mg/kg metronidazole, Sigma) in 100 µl phosphate buffered saline (PBS) 8h and 24h after CLP onset. For organ isolation experiments, a sham procedure was also performed. Here, the cecum of mice was exposed but not ligated or punctured. Mice were euthanized *via* cervical dislocation at the indicated timepoints after sepsis initiation, and plasma and organs were collected.

### 2.5 Reagents

LPS from *Salmonella abortus equi* was purchased from Sigma-Aldrich N.V. (L-5886). For *in vivo* DEX injection, Rapidexon (Medini N.V.) was used. LPS and DEX were diluted in PBS. Luciferin (XenoLight™ D-Luciferin - K+ Salt) was purchased from Caliper Life Sciences.

### 2.6 Injections and sampling

All injections were given i.p., except for the hydrodynamic intravenous (i.v.) tail injection of the DNA plasmid. Injection volumes were always adapted to the bodyweight of the mice. In lethality experiments, mice were monitored by measuring rectal body temperature. Mice with body temperature below 28°C were euthanized using cervical dislocation. Blood was taken *via* cardiac puncture after sedation of the mice with a ketamine/xylazine solution (Sigma-Aldrich N.V.) or *via* retro-orbital eye bleeding after sedation with isoflurane. To obtain mouse plasma, blood samples were collected in EDTA-coated tubes, and samples were centrifuged at 3.000 rpm for 15 minutes at 4°C. Plasma samples were stored at -20°C for biochemical analysis. For sampling of liver, mice were killed by cervical dislocation at indicated time points.

### 2.7 Hypoxia treatment

Mice were randomly assigned to the normoxia group and hypoxia group. The normoxia group was exposed to room air (21% O_2_), whereas the hypoxia group was placed in a ventilated hypoxic chamber with 7% O_2_ and 93% N_2_ for the indicated time points. The oxygen levels were monitored with a Greisinger GOX 100 oxygen sensor (Conrad).

### 2.8 Detection of HIF activity

Mice were injected in the tail vein over five seconds with a HRE-luciferase reporter plasmid solution (Addgene, #26731; 10 µg/ml in sterile, endotoxin-free PBS) or PBS (control) in a volume equivalent to 10% of the body weight, as described by Van Bogaert et al. (2011) (31). The HRE-luciferase plasmid contains three hypoxia response elements (24-mers, TGTCACGTCCTGCACGACTCTAGT) from the mouse *Pgk1* gene upstream of firefly luciferase. Five hours after transfection, mice were subjected to a sham or CLP procedure, or injected with PBS or LPS, and visualized at indicated time points. Briefly, mice were injected with 200 μl of a 15 mg/ml potassium salt luciferin solution. 10 minutes after injection, livers were isolated and visualized *via* the imaging chamber of the IVIS Spectrum *In Vivo* Imaging System (Caliper Life Sciences). Photon emission was integrated over a period of 2 minutes and recorded as pseudo-color images. Living Image (Caliper Life Sciences) was used for image analysis. The regions of interest were selected based on the luciferase signal (purple) detected over all images. To confirm the specificity of the technique used for the injection of the HRE-luciferase reporter plasmid, liver was also visualized. Data were acquired as photons/cm^2^/s and log(Y) transformed before statistical analysis. Results are normalized to the PBS control group.

### 2.9 RNA sequencing

#### 2.9.1 Liver – CLP dataset

We used liver CLP datasets GSE160795 and GSE160830 that were processed as described in Vandewalle et al. (2021) (10). Gene level read counts were obtained with featureCounts (32), and differential expressed genes were found by the DESeq2 R package (33) with the false discovery rate (FDR) set at 5%.

#### 2.9.2 Liver – Hypoxia dataset

We used liver hypoxia datasets GSE162100 and GSE162155 that were processed as described in Vanderhaeghen et al. (2021) (26). Gene level read counts were obtained with featureCounts (32), and differential expressed genes were found by the DESeq2 R package (33) with the false discovery rate (FDR) set at 5%.

### 2.10 Real-time quantitative PCR

Liver was isolated, put in RNA later (Life Technologies Europe), and stored at -20°C before RNA was isolated. Total RNA was isolated with the Aurum total RNA mini kit (Biorad) according to manufaturer’s instructions. RNA concentration was measured with the Nanodrop 8000 (Thermo Fisher Scientific), and 1000 ng RNA was used to prepare cDNA with Sensifast cDNA Synthesis Kit (Bioline). cDNA was diluted 20 times in ultrapure water for use in RT-qPCR reactions. RT-qPCR primers for used targets are listed in **Table 1**. RT-qPCR reaction was performed with sensiFast Sybr no-ROX mix (Bioline) and was performed in duplicate in a Roche LightCycler480 system (Applied Biosystems). The stability of the housekeeping genes (HKGs) were determined by Genorm. Results are given as relative expression values normalized to the geometric mean of the HKGs, calculated in the qBase+ software (Biogazelle).

**Table 1:** Primer sequences used for RT-qPCR.

Gene	Forward primer (5′‐3′)	Reverse primer (5′‐3′)
*Hprt*	AGTGTTGGATACAGGCCAGAC	CGTGATTCAAATCCCTGAAGT
*Rpl*	CCTGCTGCTCTCAAGGTT	TGGTTGTCACTGCCTCGTACTT
*Fam107a*	CAGACCAGAGTACAGAGAGTGG	GTGGTTCATAAGCAGCTCACG
*Fkbp5*	TGAGGGCACCAGTAACAATGG	CAACATCCCTTTGTAGTGGACAT
*Tsc22d3*	CCAGTGTGCTCCAGAAAGTGTAAG	AGAAGGCTCATTTGGCTCAATCTC
*Ppara*	AGAGCCCCATCTGTCCTCTC	ACTGGTAGTCTGCAAAACCAAA
*Slc25a20*	GACGAGCCGAAACCCATCAG	AGTCGGACCTTGACCGTGT
*Cpt2*	CAGCACAGCATCGTACCCA	TCCCAATGCCGTTCTCAAAAT
*Hmgcs2*	GAAGAGAGCGATGCAGGAAAC	GTCCACATATTGGGCTGGAAA
*Hif1a*	CGGCGAAGCAAAGAGTCTGAAG	GATGGTGAGCCTCATAACAGAAGC
*Epas1*	CTGAGGAAGGAGAAATCCCGT	TGTGTCCGAAGGAAGCTGATG

### 2.11 Biochemical analysis

Blood glucose and ketone body levels were measured in tail blood with the use of OneTouch Verio glucose meter (LifeScan) and Freestyle Precision Neo meter (Abbott), respectively. Free fatty acids (Abnova) were measured in mouse plasma with the use of colorimetric assays according to manufacturer’s instructions.

### 2.12 Statistics

Data were expressed as means ± standard errors of the means (SEM). Statistical significance was evaluated with a two-way ANOVA in GraphPad Prism 9.0 software (GraphPad Software, San Diego, CA). If applicable, two-way ANOVA analysis were followed by *post-hoc* analysis to correct for multiple testing during the pairwise multiple comparisons using the Šídák’s multiple comparisons test. Fold changes or ratios were log(Y) transformed before statistical analysis. Survival curves were subjected to the Log-Rank (Mantel-Cox) test to investigate whether statistical significance could be observed during different groups. Determining if two sets showed a significant overlap as approached as a (gene) set enrichment analysis, the hypergeometric test was used to obtain a p-value of the overlap. As the population size for the test, we used the total number of genes (13.000 genes) for which we can reliably obtain (normalized counts > 1 in 50% of the samples or all samples of one condition) gene level counts in the liver.

## 3 Results

### 3.1 HIF signaling is enriched in the liver during CLP-induced polymicrobial sepsis

**Figure 1:**
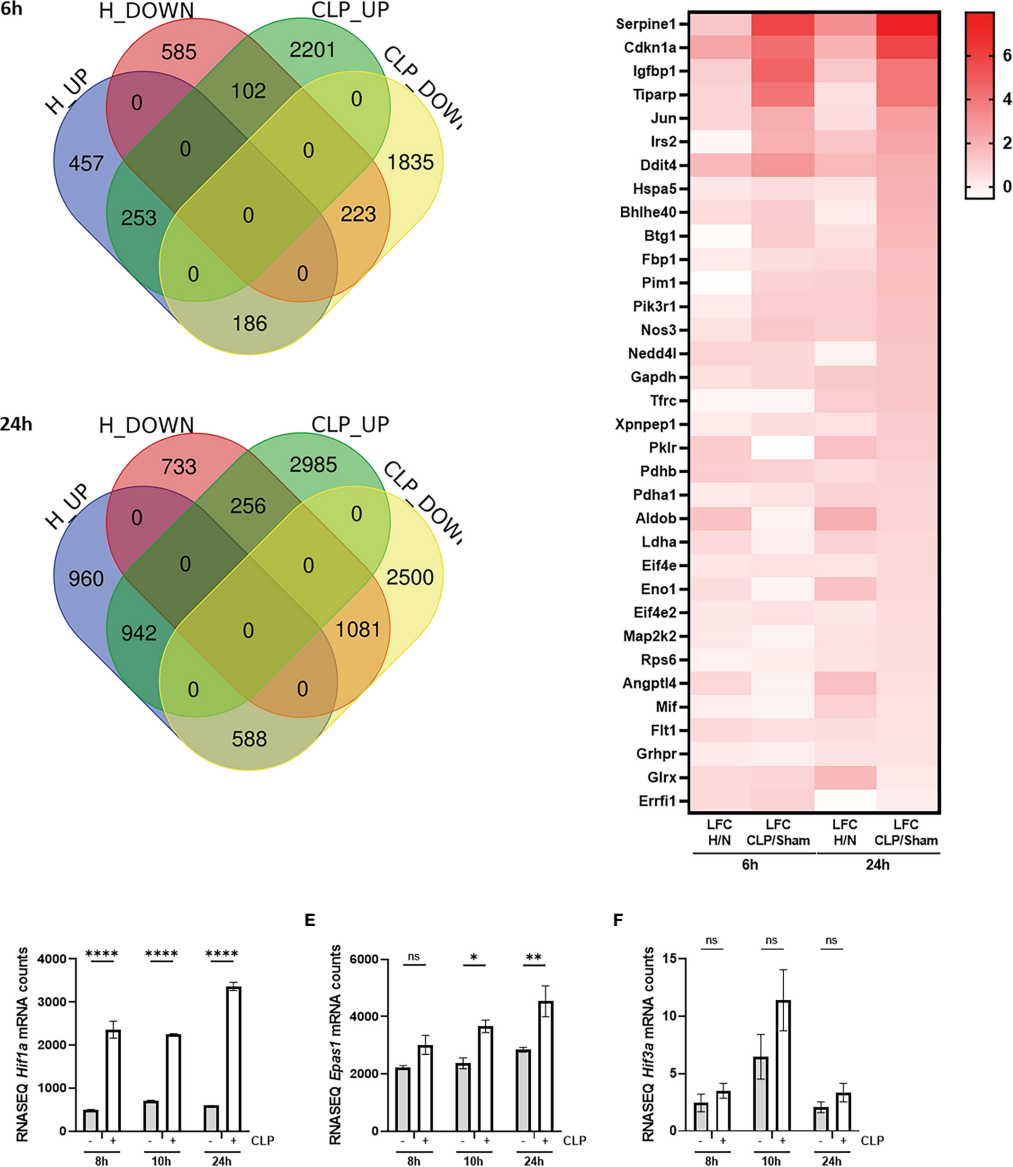
HIF signaling is enriched during sepsis. **(A, B)** C57BL/6J mice were put in normoxia or hypoxia and sham or CLP, and livers were isolated after 6h **(A)** and 24h **(B)** for genome-wide transcriptomics *via* RNA-SEQ. Venn diagram depicting the overlap between genes that are upregulated (up, P < 0.05 & LFC > 0) and downregulated (down, P < 0.05 & LFC < 0) by hypoxia and CLP at the indicated timepoints. **(C)** Heatmap based on the genes identified *via* Enrichr pathway analysis (MSigDB Hallmark 2020) significantly upregulated by CLP and by hypoxia. Log fold changes (LFCs) are depicted. **(D-F)** C57BL/6J mice were subjected to a sham or CLP procedure and the liver was isolated at the indicated timepoints. RNA-SEQ mRNA counts are shown for *Hif1a* **(D)**, *Epas1* **(E)** and *Hif3a* **(F)**. All bars represent mean ± SEM. P-values were calculated using two-way ANOVA. ****P < 0.0001, ** P ≤ 0.01; * P ≤ 0.05. ns, non-significant.

To investigate the presence of HIF signaling on a genome-wide level in the liver of septic mice, bulk RNA-SEQ analysis was performed on the livers of mice 6h and 24h after a CLP or sham procedure, and 6h and 24h after hypoxia (7% oxygen) or normoxia (**Figure 1**). After 6h, 896 and 2556 genes were significantly upregulated (adjusted P-value (P) < 0.05, LFC > 0), while 910 and 2244 genes were significantly downregulated (P < 0.05, LFC < 0) after hypoxia or CLP, respectively (**Figure 1A**). We identified the upregulation of 2490 and 4183 genes (P < 0.05, LFC > 0), and the downregulation of 2070 and 4169 genes (P < 0.05, LFC < 0) 24h after hypoxia or CLP (**Figure 1B**). The overlap between the hypoxia and CLP dataset demonstrates that there is a significant enrichment of hypoxia signaling in the up- (6h: 253/2556, P = 3.68e^-11^ and 24h: 942/4183, P = 4.7e^-12^) and downregulated (6h: 223/2244, P = 2.28e^-9^ and 24h: 1081/4169, P = 2.01e^-96^) genes in CLP-induced polymicrobial sepsis at both timepoints (**Table 2**). As expected, Enrichr analysis of the shared upregulated genes shows a clear enrichment in pro-inflammatory responses as well as hypoxia at both timepoints (**Table 3**). The log fold changes (LFCs) of genes significantly upregulated by CLP and hypoxia reported by the Enrichr analysis, are shown in the heatmap of **Figure 1C**. When analyzing the pathways induced by these genes, Enrichr revealed HIF signaling pathway, as expected, but also metabolic pathways such as glycolysis. Furthermore, the mRNA expression levels of *Hif1a* (**Figure 1D**) and *Epas1* (**Figure 1E**), the genes encoding HIF1α and HIF2α respectively, are significantly higher after CLP. In contrast, the dominant-negative regulator of the HIF pathway HIF3α (34), encoded by *Hif3a*, is hardly expressed in the liver of mice isolated after CLP or sham (**Figure 1F**). In contrast, the impact of deep hypoxia on the transcriptional levels of *Hif1a*, *Epas1* and *Hif3a* is quite minimal. After 6h and 24h of hypoxia, we detected a small (but non-significant) increase in *Hif1a* mRNA expression levels of 6% and 15%, respectively. *Epas1* mRNA levels did not increase at both time points. Also in the presence of hypoxia, *Hif3a* is hardly expressed in the liver of these mice (data not shown). 

**Table 2:** The presence of hypoxia signaling after CLP-induced polymicrobial sepsis.

	% H genes present in CLP 6h	P-value	% H genes present in CLP 24h	P-value
**UPREGULATED**	253/896 (28.2%)	3.68e^-11^	942/2490 (37.8%)	4.7e^-12^
	**% of these genes in CLP**		**% of these genes in CLP**	
	253/2556 (9.9%)		942/4183 (22.5%)	
	**% H genes present in CLP**		**% H genes present in CLP**	
**DOWNREGULATED**	223/910 (24.5%)	2.28e^-9^	1081/2070 (52.2%)	2.01e^-96^
	**% of these genes in CLP**		**% of these genes in CLP**	
	223/2244 (9.9%)		1081/4169 (25.9%)	

**Table 2** displays the amount of genes that are upregulated (adjusted P-value (P) < 0.05 and LFC > 0) or downregulated (P < 0.05 and LFC < 0) by hypoxia and how many of these genes are induced (P < 0.05 and LFC > 0) or repressed (P < 0.05 and LFC < 0) by CLP polymicrobial sepsis. Determining if two sets showed a significant overlap as approached as a (gene) set enrichment analysis, the hypergeometric test was used to obtain a p-value of the overlap. As the population size for the test, we used the total number of genes (13.000 genes) for which we can reliably obtain (normalized counts > 1 in 50% of the samples or all samples of one condition) gene level counts in the liver. By using a hypergeometric test, a significant enrichment of hypoxia signaling is present during CLP-induced sepsis.

**Table 3:** Enrichr analysis of upregulated genes shared between hypoxia and CLP.

Hypoxia vs CLP 6h		Hypoxia vs CLP 24h	
Name	P-value	Name	P-value
TNFα Signalling ***via*** NFκB	3.967e-9	Myc Targets V1	8.887e-15
Unfolded Protein Response	0.002106	Unfolded Protein Response	4.334e-11
Hypoxia	0.002657	mTORC1 Signalling	4.807e-9
Estrogen Response Early	0.002657	Protein Secretion	0.00002675
TGF-beta Signalling	0.002657	Adipogenesis	0.00008132
Inflammatory Response	0.007646	Hypoxia	0.02021
Myogenesis	0.01975	p53 Pathway	0.02021
mTORC1 Signalling	0.01975	Myc Targets V2	0.02859
p53 Pathway	0.01975	TNFα Signalling *via* NFκB	0.02859
Myc Targets V2	0.02735	Oxidative Phosphorylation	0.02859

MSigDB Hallmark 2020 analysis of the genes that are upregulated both by hypoxia and CLP after 6h and 24h. P-values shown are the adjusted p-values provided *via* Enrichr.

### 3.2 HIF activity is detected in the liver of transgenic HIF reporter mice in CLP

**Figure 2:**
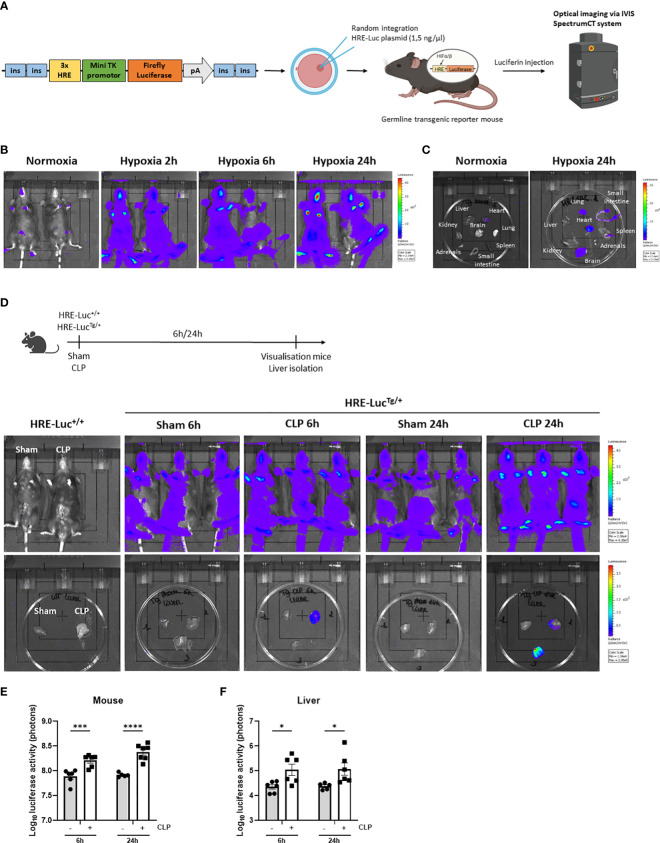
HIF activity is detected in the liver of transgenic HIF reporter mice in CLP. **(A)** A hypoxia reporter plasmid was purchased from Addgene (plasmid #26731). The plasmid contained a cassette containing three Hypoxia Responsive Elements (HRE) derived from the *Pgk1* gene (sequence HRE: TGTCACGTCCTGCACGACTCTAGT), followed by a mini TK promoter (27), firefly luciferase cDNA and SV40 polyA was flanked by 2 chicken beta-globin HS4 insulator core sequences (28) on both sides. The reporter plasmid is reported to be specific for hypoxia signals (27). The purified fragment (1.5 ng/µl) was injected in C57BL/6J zygotes and randomly integrated. Luciferase activity can be measured *via* optical imaging using the IVIS SpectrumCT system after i.p. injection of germline transgenic reporter mice and wild-type littermates with luciferin. **(B, C)** Imaging of the luciferase activity (purple signal) of HRE-Luc^Tg/+^ mice **(B)** and their organs **(C)** in normoxia and hypoxia at indicated timepoints. **(D)** Experimental set-up and imaging of the luciferase activity of HRE-Luc^Tg/+^ mice and wild-type littermates and their livers subjected to a sham or CLP procedure at the indicated timepoints. **(E, F)** Log_10_ of the bioluminescent photon counts of HRE-Luc^Tg/+^ mice **(E)** and their liver **(F)** 6h and 24h after sham or CLP (n=6/group). All bars represent mean ± SEM. Each individual data point represents individual mice. P-values were calculated using two-way ANOVA. ****P < 0.0001, ***P < 0.001, *P ≤ 0.05.

Based on the RNA-SEQ analysis, HIF signaling is present in the liver of septic mice. We have generated an HRE-luciferase reporter mouse. A hypoxia reporter plasmid was purchased from Addgene (plasmid #26731). This cassette containing 3 HREs, followed by a mini TK promotor (27), firefly luciferase cDNA, and SV40 polyA, flanked by chicken insulator sequences (28) was injected in C57BL/6J zygotes (1.5 ng/µl) and inserted *via* random integration (29). Several founder lines were obtained in which the construct had integrated. The function of the HRE-luciferase activity was measured in the germline transgenic reporter mice *via* luciferin injection and optical imaging using the IVIS SpectrumCT system as a proof-of-concept (**Figure 2A**). Heterozygous HRE-luciferase transgenic reporter (HRE-Luc^Tg/+^) mice were put in hypoxia (7% oxygen) or normoxia, and visualized after 2h, 6h and 24h. We were able to detect a clear luciferase signal under hypoxic conditions, while only a limited amount of luciferase activity was observed in normoxia (**Figure 2B**). Furthermore, the luciferase signal could be detected in several organs such as the brain, the heart and lungs, isolated from the mice after 24h of hypoxia (**Figure 2C**).

Next, using the HRE-Luc^Tg/+^ reporter mice, luciferase signals were investigated after CLP-induced polymicrobial sepsis. Therefore, a CLP or sham procedure was performed on HRE-Luc^Tg/+^ mice and wild-type littermates (HRE-Luc^+/+^). In the latter mice, no signal was observed (**Figure 2D**). A significant increase in luciferase reporter activity was detected after 6h and 24h of CLP when imaging the entire animals and their livers compared to sham-operated mice (**Figures 2D–F**), strongly suggesting that hypoxia signaling is present in the livers of septic mice and that HIF proteins are transcriptionally active in sepsis.

### 3.3 Hepatocyte-specific knock-out of HIF1α and HIF2α reduces HIF activity in the liver of septic mice

In order to confirm whether HIF1α and/or HIF2α is/are responsible for the luciferase signal detected in the liver after CLP, mice with a conditional knock-out of HIF1α (HIF1a^AlbKO^) or HIF2α (HIF2a^AlbKO^), or both (HIF1aHIF2a^AlbKO^) in hepatocytes were generated. To validate the hepatocyte specific knock-out mice used in these experiments, *Hif1a* and *Epas1* mRNA levels were measured *via* RT-qPCR in the liver of HIF1a^AlbKO^, HIF2a^AlbKO^, HIF1aHIF2a^AlbKO^ mice and wild-type littermates (**Supplementary Figure 1**). As expected, *Hif1a* and *Epas1* mRNA levels were significantly downregulated in the respective knock-out mice (**Supplementary Figures 1A, B**). Both genes were significantly downregulated in the liver of HIF1aHIF2a^AlbKO^ mice (**Supplementary Figure 1C**). We also detected a downregulation of *Epas1* mRNA in the liver of HIF1a^AlbKO^ mice (**Supplementary Figure 1A**), suggesting that HIF1α depletion also causes some HIF2α reduction under normoxic conditions.

**Figure 3:**
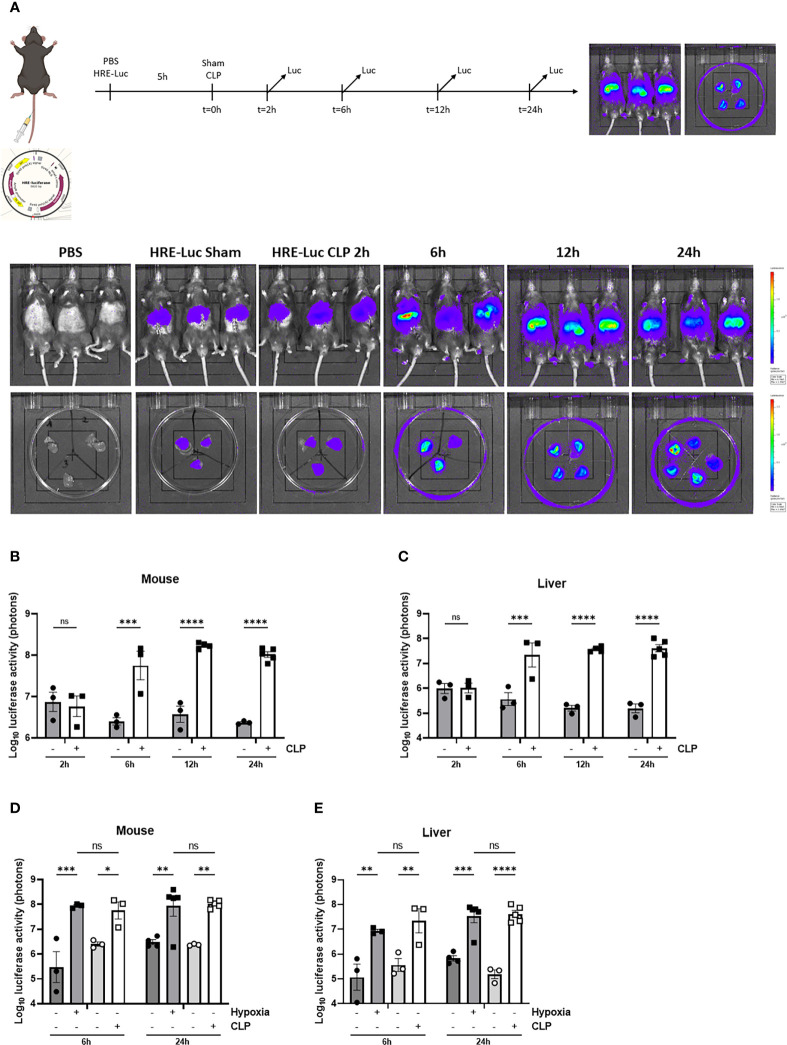
HIF activity in mouse liver during sepsis using high-pressure injections. **(A-C)** The effect of CLP on HIF activity was estimated in C57BL/6J mice by a HRE-luciferase reporter plasmid at indicated time points. All mice were injected according to body weight. **(A)** Experimental set-up and imaging of the luciferase activity (purple signal) in the liver of PBS control mice and mice after a sham or CLP procedure at the indicated time points. Log_10_ of the bioluminescent photon counts normalized to the PBS control group of C57BL/6J mice **(B)** and their livers **(C)** subjected to a sham or CLP procedure at the indicated timepoints (n=3-5/group). Log_10_ of the bioluminescent photon counts of mice **(D)** and their livers **(E)** subjected to normoxia (black circles) or hypoxia (black squares) and sham (white circles) or CLP (white squares) at the indicated time points (n=3-5/group). All bars represent mean ± SEM. Each individual data point represents individual mice. P-values were calculated using two-way ANOVA. **P ≤ 0.01; *P ≤ 0.05; ***P <0.001; ****P <0.0001. ns, non-significant.

First, we confirmed the presence of hepatic HIF activity in septic mice by injecting the HRE-luciferase reporter plasmid (which was used to generate the transgenic reporter mice) or PBS (control) *via* the tail vein under high pressure, leading to hepatocyte-specific transfection. These mice were randomly assigned to a sham or CLP procedure. Luciferase activity was measured at the indicated timepoints (**Figure 3A**). In sham mice, low luciferase signals were detected in mice injected with the reporter plasmid. As soon as 6h after CLP, the luciferase activity strongly increased and remained high until 24h post-surgery (**Figures 3B, C**), suggesting strong HIF transcriptional activity. Furthermore, we compared HRE-luciferase activity between CLP and LPS-induced endotoxemia. Mice were injected with the reporter plasmid *via* the tail vein followed by an LPS injection or a CLP procedure. A sham operation or PBS injection was performed as a control. The luciferase activity tended to increase to the same extent in both mouse models of systemic inflammatory response syndrome (SIRS) and sepsis (**Supplementary Figure 2**). Finally, we studied if the HRE-luciferase activity induced by sepsis is comparable with mice in hypoxia (7% oxygen). Therefore, mice were put in normoxic or hypoxic conditions, or were subjected to a sham or CLP procedure after high-pressure injection of the HRE-luciferase reporter plasmid. 6h after CLP, the HRE-luciferase signal was significantly increased compared to sham, remained high until 24h, and was comparable to the signal induced by hypoxia (**Figures 3D, E**). In contrast to their mRNA expression levels, HIF proteins do accumulate in hypoxic conditions. 

**Figure 4:**
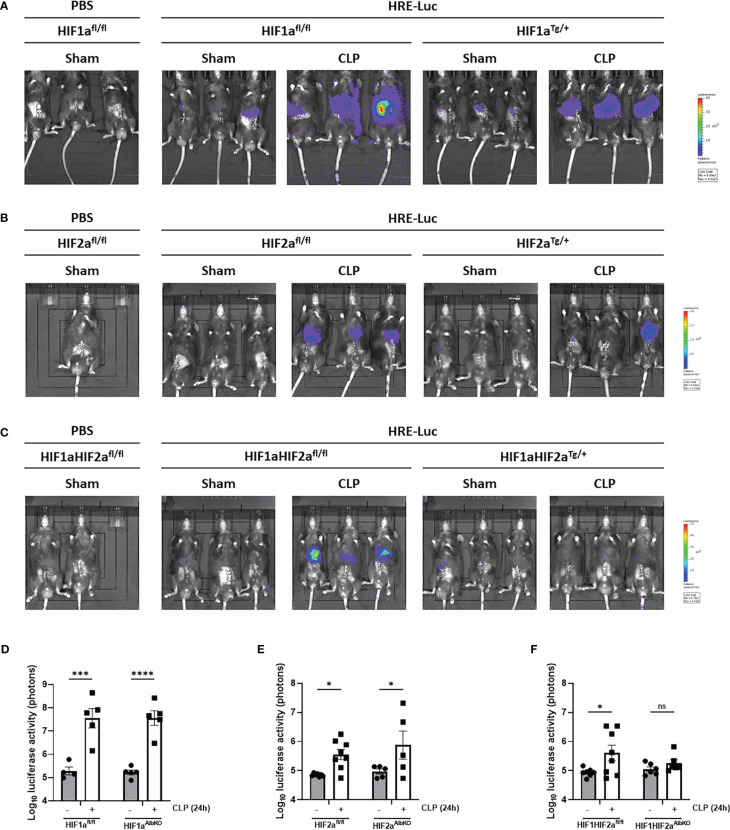
HIF activity in hepatocyte-specific knock-out mice of HIF1α and/HIF2α in sepsis. HIF1a^AlbKO^, HIF2a^AlbKO^ and HIF1aHIF2a^AlbKO^ mice and wild-type littermates were injected with the HRE-luciferase reporter mice *via* high pressure injection at the tail vein. Then, mice were subjected to a sham or CLP procedure and the luciferase activity was measured 24h post-surgery. **(A-C)** Imaging of the luciferase activity (purple signal) of HIF1a^AlbKO^ **(A)**, HIF2a^AlbKO^ **(B)** and HIF1aHIF2a^AlbKO^ **(C)** mice and wild-type littermates 24h after sham or CLP procedure. **(D-F)** Log_10_ of the bioluminescent photon counts of HIF1a^AlbKO^ **(D)**, HIF2a^AlbKO^ **(E)** and HIF1aHIF2a^AlbKO^ **(F)** mice and wild-type littermates 24h after sham or CLP procedure (n=4-8/group). All bars represent mean ± SEM. Each individual data point represents individual mice. P-values were calculated using two-way ANOVA. ****P < 0.0001, ***P < 0.001, *P ≤ 0.05. ns, non-significant.

To investigate which HIF protein is involved in the HRE-luciferase activity observed during sepsis, we measured the reporter activity in the liver of HIF1a^AlbKO^, HIF2a^AlbKO^ and HIF1aHIF2a^AlbKO^ 24h after CLP-induced polymicrobial sepsis using the reporter plasmid (**Figure 4**). The HRE-luciferase activity increased in HIF1a^AlbKO^ (**Figures 4A, D**) and HIF2a^AlbKO^ (**Figures 4B, E**) 24h after CLP, to the same extent as in wild-type mice. However, when both HIF proteins are absent in hepatocytes, we were no longer able to detect a significant increase in the HRE-luciferase activity in sepsis (**Figures 4C, F**), suggesting that both HIF1α and HIF2α are responsible for the HIF activity in sepsis and that perhaps both proteins can functionally compensate for the loss of the other in hepatocytes.

### 3.4 Survival of hepatocyte-specific knockouts of HIF1α, HIF2α or both in CLP-induced polymicrobial sepsis or LPS-induced endotoxemia

**Figure 5:**
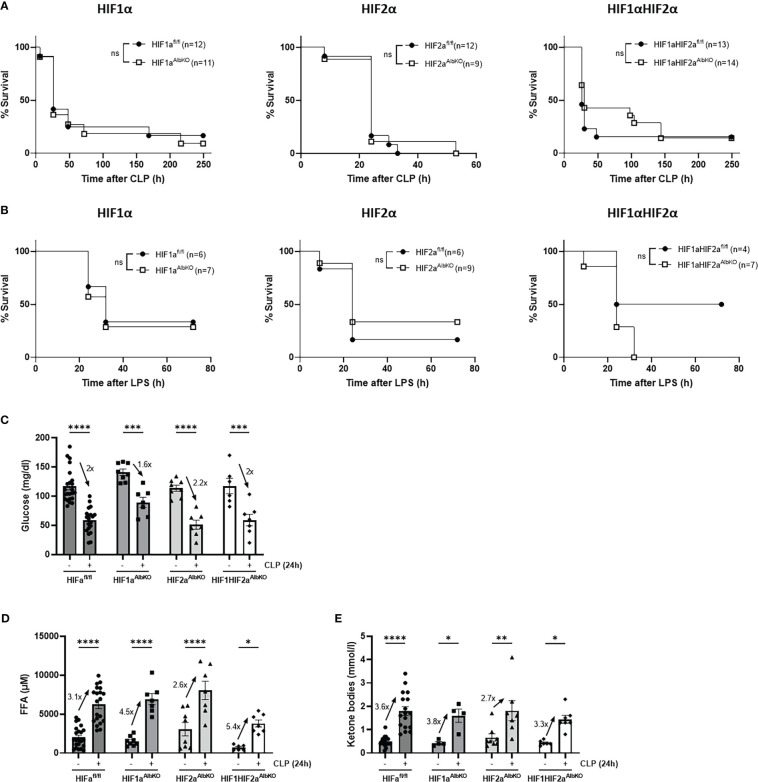
Lack of survival benefit in CLP-induced polymicrobial sepsis or LPS-induced endotoxemia in the absence of HIF1α and/or HIF2α in hepatocytes. HIF1a^AlbKO^, HIF2a^AlbKO^, and HIF1aHIF2a^AlbKO^ mice and wild-type littermates were subjected to CLP **(A)** or injected with 11.25 mg/kg LPS **(B)**. Survival was monitored over time. N-values are indicated in the figure. Survival curves were analyzed with Log-Rank test. **(C-E)** HIF1a^AlbKO^, HIF2a^AlbKO^, and HIF1aHIF2a^AlbKO^ mice and wild-type littermates were subjected to a sham or CLP procedure. After 24h, glucose **(C)** and ketone bodies **(E)** were measured *via* the tail vein. FFA levels **(D)** were determined in the plasma of these mice. Fold inductions are shown on the figures. All bars represent mean ± SEM. P-values were analyzed with two-way ANOVA. ****P < 0.0001, ***P < 0.001, **P < 0.01, *P ≤ 0.05. ns, non-significant.

Several studies have shown that a conditional HIF1α or HIF2α knock-out in myeloid cells protects against LPS-induced endotoxemia (35–37). However, the role of hepatic HIF proteins in polymicrobial sepsis has been poorly studied. Therefore, we first investigated whether HIF1α and/or HIF2α expression in the liver contribute to sepsis mortality. Mice with a conditional knock-out of HIF1α (HIF1a^AlbKO^) or HIF2α (HIF2a^AlbKO^), or HIF1α and HIF2α (HIF1aHIF2a^AlbKO^) in hepatocytes and wild-type littermates were subjected to a CLP procedure. However, no survival benefit was observed in the absence of HIF1α and/or HIF2α in hepatocytes after CLP (**Figure 5A**). Furthermore, when these mice were injected with a lethal dose of LPS (11.25 mg/kg), no significant impact on survival between hepatocyte-specific HIFa^AlbKO^ mutant and wild-type mice was observed (**Figure 5B**).

As mentioned before, sepsis is characterized by a (failing) SR with hypoglycemia and increased levels of FFAs and KBs as a consequence (7, 8). Furthermore, HIF proteins are involved in regulating the expression of genes involved in glucose and lipid metabolism (21–23) in a direct way, or by reducing the function of PPARα and/or GR in hepatocytes (hypothesis investigated in this study). Therefore, we investigated whether the absence of HIF1α and/or HIF2α in hepatocytes has an influence on the hypoglycemia and increased FFA and KB levels during sepsis. HIF1a^AlbKO^, HIF2a^AlbKO^ and HIF1aHIF2a^AlbKO^ mice and wild-type littermates were subjected to a CLP or sham procedure and glucose, FFAs and KBs were measured in the blood of these mice 24h post-surgery. As expected (10), hypoglycemia was detected in wild-type mice 24h after CLP. The degree of hypoglycemia was similar in all three mutant mice (**Figure 5C**). We could also detect a significant increase in both FFA and KB levels in the blood of wild-type mice and all three HIFa^AlbKO^ mice, 24h after polymicrobial sepsis was induced (**Figures 5D, E**). Although some differences in the degree of hypoglycemia, and FFA and KB increases were detected in the different groups, we conclude that, by and large, no major effects of HIF absence were found on biological effects of CLP-induced hypoglycemia and increase in FFA and KB levels.

### 3.5 Hepatic HIF1α and HIF2α are not involved in the GCR present in polymicrobial sepsis

**Figure 6:**
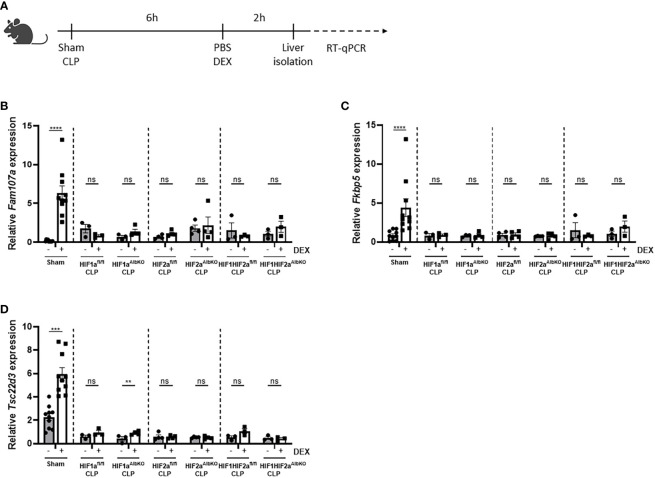
No role for HIF1α and/or HIF2α in hepatocytes in mediating GCR during sepsis. **(A)** HIF1a^AlbKO^, HIF2a^AlbKO^ or HIF1aHIF2a^AlbKO^ mice and wild-type littermates were randomly assigned to a sham or CLP procedure. 6h post-surgery, mice were injected i.p. with PBS or DEX (10 mg/kg) and the livers were isolated 2h later. The mRNA expression levels of *Fam107a* **(B)**, *Fkbp5* **(C)** and *Tsc22d3* **(D)** were measured *via* RT-qPCR. One experiment (n=3/group). Data is pooled for the wild-type mice. All bars represent mean ± SEM. P-values were analyzed *via* two-way ANOVA. ****P < 0.0001, ***P < 0.001. ns, non-significant.

Once polymicrobial sepsis *via* CLP is induced, mice develop a persistent and genome-wide GCR in the liver as well as hypoglycemia and hyperlactatemia (10). Furthermore, since HIFs are thought to be involved in increased glycolysis thereby contributing to the higher blood lactate levels during sepsis (7), and a clear crosstalk exists between GR and HIF (25), we investigated if HIF1α and/or HIF2α are involved in the GCR induced in the liver during sepsis. Therefore, HIF1a^AlbKO^, HIF2a^AlbKO^ or HIF1aHIF2a^AlbKO^ mice and wild-type littermates were subjected to a CLP procedure. After 6h, a timepoint at which GCR is already present in mice (10), mice were injected i.p. with PBS or DEX (10 mg/kg), and liver was isolated 2h later (**Figure 6A**). The expression of typical GR-responsive genes was measured *via* RT-qPCR. In sham mice, a significant increase in the mRNA expression levels of *Fam107a*, *Fkbp5* and *Tsc22d3* after DEX stimulation was detected. As previously shown (10), these genes no longer responded to DEX after CLP. Furthermore, we were unable to detect any significant difference in gene expression levels after DEX stimulation in the liver of HIF1a^AlbKO^, HIF2a^AlbKO^ and HIF1aHIF2a^AlbKO^ mice 6h after CLP (**Figures 6B–D**). These results show that the absence of HIF1α and/or HIF2α in hepatocytes of septic mice is not able to prevent GCR during sepsis.

### 3.6 Effect of HIF1α and HIF2α on PPARα functioning in sepsis

**Figure 7:**
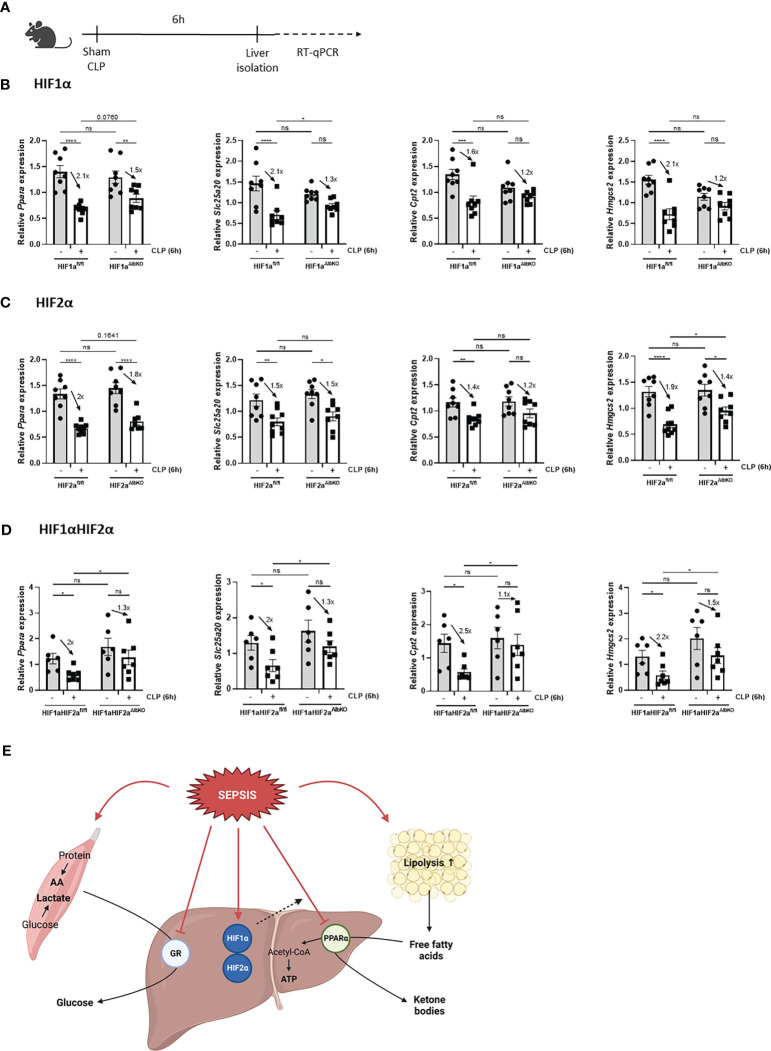
Absence of HIF1α in hepatocytes might affect impaired PPARα signaling during sepsis. HIF1a^AlbKO^, HIF2a^AlbKO^ or HIF1aHIF2a^AlbKO^ mice and wild-type littermates were randomly assigned to a sham or CLP procedure. 6h post-surgery, livers were isolated. **(A)** Experimental set-up. **(B-D)** The expression levels of *Ppara* and PPARα responsive genes were measured in the liver of HIF1a^AlbKO^ (n=8/group) **(B)**, HIF2a^AlbKO^ (n=8-9/group) **(C)**, or HIF1aHIF2a^AlbKO^ (n=6-7/group) **(D)** mice and wild-type littermates *via* RT-qPCR. **(E)** Graphical abstract. Fold inductions are displayed on the graphs. All bars represent mean ± SEM. P-values were analyzed with two-way ANOVA. ****P < 0.0001, ***P < 0.001, **P < 0.01, *P ≤ 0.05. ns, non-significant.

Sepsis is also associated with a rapid decline in hepatic PPARα mRNA and protein levels, and hence a reduced hepatic FFA β-oxidation catabolism. In combination with increased lipolytic activity of WAT, this reduced β-oxidation causes lipotoxicity in liver and kidney after sepsis (11). Since hypoxia is associated with increased lipolysis, increased FFA levels in the blood (26), and impaired FFA β-oxidation (22, 23), and p300 functions as a co-activator for PPARα (38), hepatic HIF1α and/or HIF2α might be involved in the declined PPARα signaling during sepsis. Therefore HIF1a^AlbKO^, HIF2a^AlbKO^, and HIF1aHIF2a^AlbKO^ mice and wild-type littermates were subjected to CLP and livers were isolated 6h later (**Figure 7A**). In wild-type mice, a rapid decline of *Ppara* and several PPARα responsive genes was observed 6h after CLP (**Figures 7B–D**). In the absence of HIF1α in hepatocytes, the mRNA expression levels of *Ppara* and its responsive genes were decreased, although less pronounced than in wild-type littermates (**Figure 7B**). This might indicate that HIF1α contributes to the decline in PPARα and its signaling in sepsis. In the absence of HIF2α in hepatocytes, no major effect was observed on the expression of *Ppara* and PPARα responsive genes 6h after a CLP procedure in comparison to wild-type mice (**Figure 7C**). In line with the results obtained in HIF1a^AlbKO^ mice, the expression levels of *Ppara* and PPARα responsive genes were significantly reduced in the liver of HIF1aHIF2a^AlbKO^ mice and its wild-type littermates 6h after sepsis. However, the downregulation of *Ppara* and its targets genes is also less pronounced in the liver of HIF1aHIF2a^AlbKO^ mice (**Figure 7D**).

Altogether, we conclude that HIF proteins are not involved in the appearance of GCR in liver during sepsis and that HIF1α in hepatocytes of septic animals might be involved in the reduced PPARα signaling (**Figure 7E**).

## 4 Discussion

Polymicrobial sepsis is a systemic disease, affecting several organ systems. Nevertheless, certain organs are crucial in the progression of sepsis. Within the context of the lack of food intake in sepsis and the consequent SR, the liver is confronted with high levels of FFAs, glycerol and gluconeogenic AAs, which require PPARα and GR, respectively, to be properly transformed into acetyl-CoA, KBs, and glucose (8). The acute lack of PPARα and GR function during sepsis makes the liver key in sepsis (10, 11), not only because this organ produces acute phase proteins (39–41). So, the investigation of the mechanistic aspects of these failures of transcription factors is really essential. Because crosstalk of transcription factors in physiology and pathology is a commonly observed phenomenon, we hypothesized that HIF1α and/or HIF2α might play a role in the PPARα and GR dysfunction during sepsis. To show, unambiguously that HIF factors are activated in liver in sepsis, we applied the CLP model, considered as being the best validated model of polymicrobial (peritoneal) sepsis in mice. Furthermore, we have generated a transgenic HIF-luciferase reporter mouse by using the commercial HRE-luciferase reporter construct, which has been validated as being a tool specific for HIF factors (27).

Inflammation and hypoxia are unequivocally linked (42). Just as hypoxia causes inflammation, inflamed tissue can become severely hypoxic (43). Dynamic changes in (protein) HIF expression occur during sepsis and therefore studies have proposed HIFs as potential biomarkers or important players in sepsis, however results are often controversial. Transcriptome data from peripheral blood mononuclear cells (PBMCs) obtained from sepsis and septic shock patients have shown that hypoxia and glycolysis were among the top scored molecular signatures. Furthermore, the expression of HIF1α and its target genes were higher in non-survivors sepsis patients compared to survivors (44). In experimental models, HIF1α plays an important role in the bactericidal capacity of macrophages to prevent systemic spreading of an infection (45–47), and conditional knock-out of HIF1α or HIF2α in myeloid cells protects mice against LPS-induced endotoxemia by reducing the pro-inflammatory cytokine production, hypothermia, and hypotension (35–37). Also dimethyloxalylglycine (DMOG), a PHD inhibitor leading to HIF1α stabilization, increased the survival of mice against LPS-induced endotoxemia, however it exacerbated disease severity in polymicrobial sepsis (48). HIF1α also has a vital role in the initial metabolic shift from oxidative phosphorylation to glycolysis during sepsis (49), and attenuates the pro-inflammatory response by inducing IRAKM production, a negative regulator of TLR signaling (50). Taken together, these studies suggest an important role for HIF1α during various stages of sepsis, despite most animal experiments applied suboptimal sepsis model systems, like the LPS-induced endotoxemia mouse model.

Besides this (controversial and incomplete) information from literature, data from our research group urged us to study the role of HIF proteins in hepatocytes during sepsis. Next to a reduced GR DNA-binding profile in CLP mice, we have demonstrated that TNF-mediated GCR could be a result of the sequestration of co-factor p300 to NF-κB, thereby preventing its accessibility to GR (15). P300 is known as a histone acetyltransferase (HAT) essential for GR-mediated transcription (51), but also for other transcription factors such as NF-κB and HIF (52). For example, lysine acetyltransferase 5 (KAT5) and cAMP response element binding protein (CBP)/p300 acetylate histones at HIF bound loci and are required for the transcriptional activation of a subset of HIF target genes (53, 54). Recently, it has been shown that hypoxia differentially regulates H3K27 acetylation at GR binding sites (55). The p300 co-activator is responsible for the increased histone acetylation and the increased recruitment of GR to its DNA binding sites (15, 56). Moreover, we and others have shown that there is a clear interaction between HIF and GR (26, 57, 58). Therefore, a competition between GR and HIF for the p300 co-activator might thus be responsible for the alterations in the gene expression profile. Next to GCR, sepsis is also characterized by a PPARα dysfunction in the liver (11, 16). Following ligand binding, the AF-2 domain of PPARα undergoes conformational changes, which allows the interaction of several co-factors such as CBP/p300 (38, 59). Therefore, we wanted to investigate whether HIF1α and HIF2α expression in hepatocytes contributes to the GCR and PPARα failure present in sepsis, as this has not been studied before.

In our studies, bulk RNA-SEQ performed 6h and 24h after onset of a lethal polymicrobial sepsis in mice, convincingly proved that HIF transcription factors are upregulated on the mRNA level, and also lead to a significant accumulation of HIF-dependent transcripts. Using the HRE-luciferase transgenic reporter mice that were generated for this study, HIF activity was detected in the livers of CLP mice 6h post-surgery and remained high until 24h after surgery. Based on the HRE-luciferase reporter activity measured in HIFa^AlbKO^ mice, both HIF1α and HIF2α appear responsible for the HIF activity, the signals of which are only annihilated when both HIF1α and HIF2α are knocked out in the hepatocytes. Based on the reporter plasmid (27), HIF activity was similar in mouse models for endotoxemia and sepsis, and could be compared to the amount of activity observed in hypoxia. Unfortunately, although we were able to detect HIF activity in the liver during sepsis, we were unable to observe a survival benefit in LPS-induced endotoxemia and CLP polymicrobial sepsis in mice lacking HIF1α and/or HIF2α in hepatocytes. There are two possible explanations for this observation. Either, HIF factors are activated in hepatocytes, but play no mechanistic role in sepsis, or the transcriptional signals and reporter activities that were observed in the liver were from cells other that hepatocytes. Next to hepatocytes, which form 70% of the cells in the liver, 10% of the liver cells are Kupffer cells (KCs) (60). Also liver sinusoidal endothelial cells (LSECs, 15%) and hepatic stellate cells (HSCs, 5%) cooperate to shape and maintain liver function (61) and could thus be involved in the HIF activity observed in the liver during sepsis. For follow-up studies, it might also be considered to test the effect of the absence of HIF proteins in hepatocytes in more slowly progressive models of sepsis such as a systemic *Staphylococcus aureus* or *Klebsiella pneumoniae* infection (62).

As mentioned earlier, the two main transcription factors involved in the metabolic reprogramming during sepsis in the liver are the GR and PPARα, associated with hypoglycemia and hyperlactatemia (10), and increased levels of FFAs and glycerol in the blood (7, 11). Upon an infection, the immune system will protect the host by eradicating the pathogen. This is often associated with inflammation and tissue damage, which could harm the host. In order to maintain homeostasis and survive excessive inflammation, cytokines produced by the immune cells will activate the hypothalamus-pituitary-adrenal (HPA) axis and induce GC synthesis as a disease tolerance mechanism (63, 64). We have shown that hypoxia is able to stabilize HIF1α and HIF2α at the hypothalamus and stimulates the HPA axis leading to GC production (26). Furthermore, this HPA axis activation is essential for sepsis survival, since surgical removal of the pituitary or adrenal glands (65), or pharmacological inhibition of GR by RU486 (66) sensitizes mice to sepsis. The GCs produced regulate the disease severity by dampening the inflammatory responses *via* monomeric GR-mediated tethering to transcriptional factors NF-κB and AP-1 (67). Also the GR dimer is important, because GR^dim/dim^ mice are more sensitive and are unable to induce a proper inflammatory response in the absence of proper GR dimerization (68–71). *In vivo* studies using zebrafish and mice have shown that the upregulation of HIF signaling alters the GR activity and dampens its responsiveness to GR agonists such as DEX and betamethasone (26, 55, 58). Furthermore, GCs are essential for hepatic gluconeogenesis to provide sufficient glucose levels (72). Increased glycolytic activity is associated with an increased conversion of pyruvate into lactate during sepsis (73). Due to GCR, lactate-based gluconeogenesis in the liver, also known as the Cori cycle, is inhibited in sepsis (10). In addition, HIF1α also stimulates the expression of glycolytic genes, which further contributes to the conversion of pyruvate into lactate (74). Therefore, we wanted to investigate the involvement of hepatocyte-specific expression of HIF1α and/or HIF2α in the GCR observed during sepsis, however without any positive results.

Next to GCR, sepsis is also characterized by a PPARα dysfunction in the liver. This PPARα dysfunction can, in part, be explained by rapid decline of hepatic PPARα mRNA and protein levels and activity, which leads to a reduced expression of its target genes involved in FFA β-oxidation (11, 16). Since sepsis acutely activates lipolysis in WAT, increased FFA and glycerol levels are present in the blood of sepsis patients (75–77). As a consequence of PPARα malfunction, FFAs are no longer oxidized which leads to the ectopic deposition of lipid storages in liver and kidney after sepsis and thereby causes lipotoxicity and tissue damage (11, 78). Hypoxia stimulates the release of FFAs in the blood of mice in a GC/GR-dependent way (26). Since FA catabolism is altered under hypoxia, an excess of intracellularly accumulated FFAs could cause lipotoxicity. Cells try to avoid this by converting FFAs into neutral triacylglycerols (TAGs), which can be stored in lipid droplets and form the main energy depots (79, 80). HIF1α directly upregulates the expression of acylglycerol-3-phosphate acyltransferase 2 (AGPAT2) (81) and lipin-1 (82), both important for the formation of lipid droplets. Furthermore, HIF2α has been shown as a master regulator in hepatic lipid metabolism during hepatosteatosis. The absence of HIF2α, and not HIF1α, in *Vhl* knock-out mice protected against hepatic lipid accumulation (83, 84). Moreover, Rey et al. (2020) have demonstrated that HIF2α induces the expression of CD36, the major driver of FFA uptake, which triggers lipid accumulation in hepatocytes both *in vitro* and *in vivo* (85). In addition, HIF2α also increases the expression of the adipose differentiation-related protein (ADRP) in the liver (86), also involved in FFA uptake, and reduced FA β-oxidation (87). When oxygen therapy is provided, hepatic steatosis induced by high-fat diet (HFD) is ameliorated *via* the reduction of hepatic HIF2α and lipogenic gene expression (88). In general, the abovementioned studies suggest that HIF2α increases FFA uptake and *de novo* lipogenesis as well as decreases β-oxidation. Regarding HIF1α, it has been shown that systemic or hepatic *Hif1a* deletion or HIF1α antisense oligonucleotides reduces hepatosteatosis (89, 90). On the contrary, other studies revealed HIF1α mediated protection against alcohol-induced fatty liver disease (91, 92).

It has been shown that PPARα is essential for sepsis survival. PPARα knock-out mice are more susceptible to a lethal dose of LPS (93) and bacterial infections (16, 94), which is associated with increased kidney failure and heart injury (94, 95). Also, mice treated with the PPARα antagonist GW6471 are more prone to CLP-induced polymicrobial sepsis. Although hepatic PPARα plays an essential role during sepsis survival, a genome-wide disturbance of PPARα function is observed in mouse septic livers upon stimulation with the PPARα agonist GW7647 (11). Since HIF1α and HIF2α are involved in the regulation of lipid metabolism in the liver (22, 23), and the effect of hepatic HIF protein expression on the PPARα dysfunction has not been studied in sepsis, we have investigated whether the absence of HIF1α and/or HIF2α in hepatocytes of septic animals influences PPARα function. We were able to identify some promising results in the absence of HIF1α expression in hepatocytes. It would thus be of great interest to investigate whether hepatic PPARα stimulation with pemafibrate or GW7647, well known PPARα agonists, in the absence of HIF1α in hepatocytes during sepsis might be able to restore PPARα functioning and reduce lipotoxicity.

In summary, we have shown the presence of HIF signaling in the liver during CLP-induced polymicrobial sepsis using RNA-SEQ data and the HRE-luciferase reporter mice. However, hepatocyte-specific knock-out mice for HIF1α and/or HIF2α did not yield any survival benefit against LPS-induced endotoxemia and CLP polymicrobial sepsis. Since a conditional knock-out of HIF1α or HIF2α in myeloid cells protects against LPS (35, 36), it might be of interest to study whether KC specific knock-out mice for HIF1α and/or HIF2α are protected against LPS-induced endotoxemia and CLP polymicrobial sepsis. Another option could be to revisit the RNA-SEQ data in single cell populations, or in FACS-separated cell types of the liver, and equally so revisit the luciferase reporter data. Unfortunately, the absence of HIF proteins in hepatocytes is not able to prevent GCR in sepsis. Finally, we were able to identify a potential role for HIF1α in hepatocytes of septic animals in the reduced PPARα signaling.

## Data availability statement

Publicly available datasets were analyzed in this study. This data can be found here: Liver – CLP dataset We used liver CLP datasets GSE160795 and GSE160830 that were processed as described in Vandewalle et al. (2021) (10). Liver – Hypoxia dataset We used liver hypoxia datasets GSE162100 and GSE162155 that were processed as described in Vanderhaeghen et al. (2021) (26).

## Ethics statement

The animal study was reviewed and approved by Institutional ethics committee for animal welfare of the Faculty of Sciences, Ghent University, Belgium.

## Author contributions

TV conceived and performed the experiments and co-wrote the manuscript. ST performed all bio-informatics analysis of RNA sequencing. JV, CW, LN, DW performed experiments. JV performed the CLP experiment with DEX stimulation in HIF1a^AlbKO^ mice to study the GCR. LN performed the CLP experiment with DEX stimulation in HIF2a^AlbKO^ mice to study the GCR. TH generated the HRE-Luc mice. ME, SD, and JB provided general technical assistance. JT and NS provided an imaging chamber of the IVIS Spectrum *In Vivo* Imaging System. RB and CL supervised the research and co-wrote the manuscript. All authors contributed to the article and approved the submitted version.

## Supplementary material

Supplementary Figure 1
*Hif* mRNA and HIF proteins levels in hepatocyte-specific HIF1α and/or HIF2α knock-out mice. **(A-C)** Livers of HIF1a^AlbKO^ **(A)**, HIF2a^AlbKO^ **(B)**, or HIF1aHIF2a^AlbKO^ **(C)** mice and wild-type littermates were isolated and the expression levels of *Hif1a* and *Epas1* were measured *via* RT-qPCR. All bars represent mean ± SEM. P-values were analyzed with two-way ANOVA. ****P<0.0001, **P<0.01, *P ≤ 0.05.

Supplementary Figure 2 HRE-luciferase activity detected in LPS-induced endotoxemia *versus* CLP polymicrobial sepsis. **(A)** C57BL/6J mice were injected with the HRE-luciferase reporter plasmid *via* the tail vein using high-pressure injections. After incubation, mice were injected with PBS or LPS, or were subjected to a sham or CLP procedure. 6h later, livers were visualized using the IVIS SpectrumCT system. Log_10_ of the bioluminescent photon counts in liver of mice subjected to LPS or CLP and PBS and sham as control (n=3/group). All bars represent mean ± SEM. P-values were analyzed with two-way ANOVA. **P<0.01.
