# Supplementary figures and images for: The impact of hepatocyte-specific deletion of hypoxia-inducible factors on the development of polymicrobial sepsis with focus on GR and PPARα function
Source: Front Immunol. 2023 Mar 16;14:1124011. doi: 10.3389/fimmu.2023.1124011 (PMC10060827; doi:10.3389/fimmu.2023.1124011)

Supplementary Figure 1

A

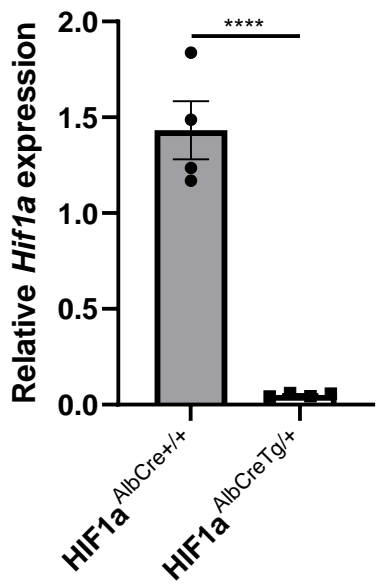

B

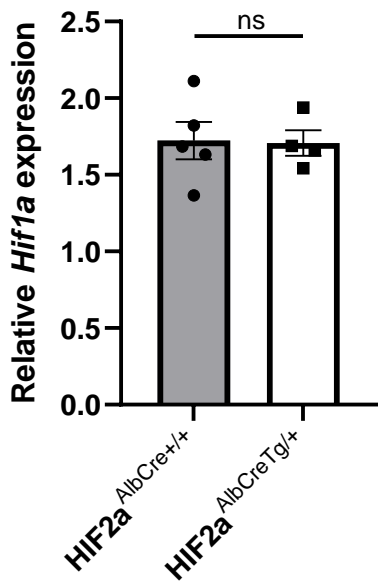

C

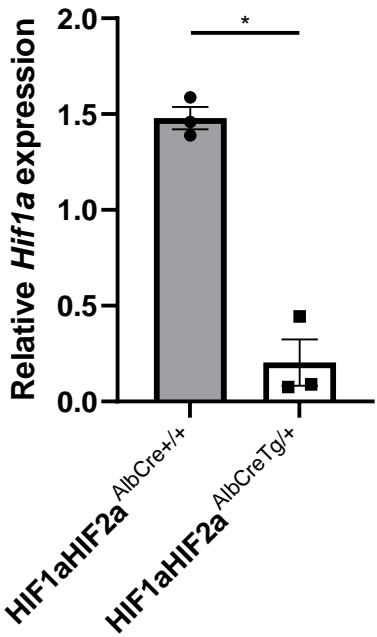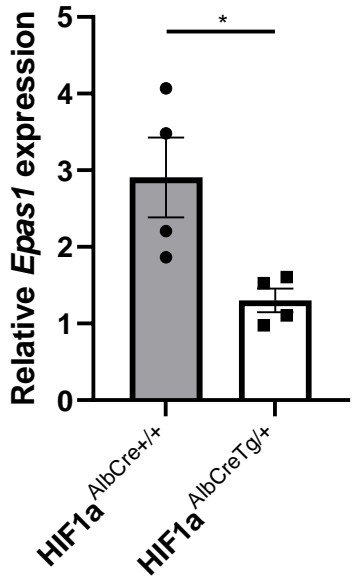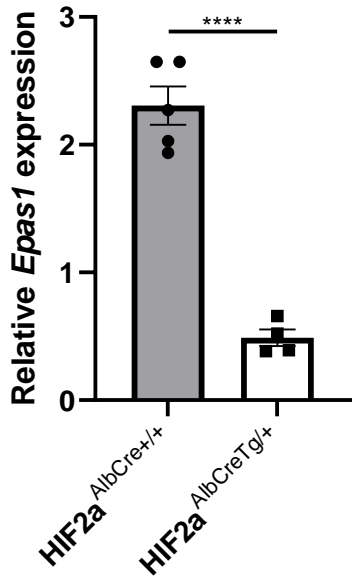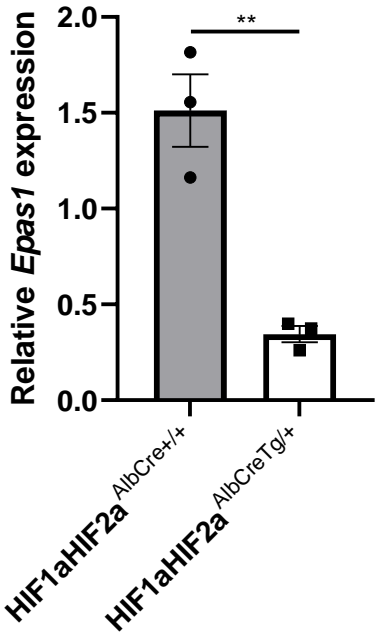

Supplement: Supplementary Figure 1 — Hif mRNA and HIF proteins levels in hepatocyte-specific HIF1α and/or HIF2α knock-out mice. (A-C) Livers of HIF1aAlbKO (A), HIF2aAlbKO (B), or HIF1aHIF2aAlbKO (C) mice and wild-type littermates were isolated and the expression levels of Hif1a and Epas1 were measured via RT-qPCR. All bars represent mean ± SEM. P-values were analyzed with two-way ANOVA. ****P<0.0001, **P<0.01, *P ≤ 0.05. [file Image_1.pdf]

Supplementary Figure 2

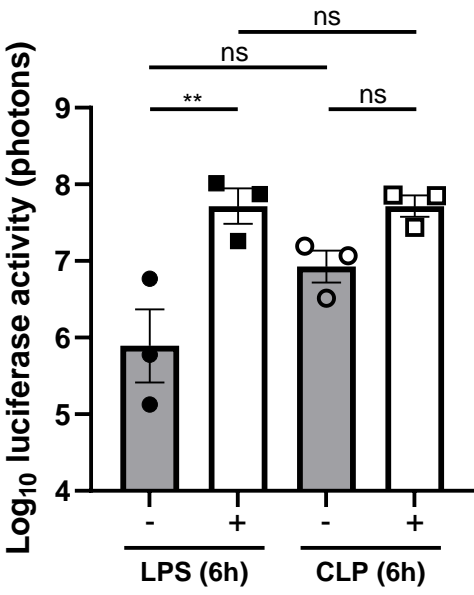

Supplement: Supplementary Figure 2 — HRE-luciferase activity detected in LPS-induced endotoxemia versus CLP polymicrobial sepsis. (A) C57BL/6J mice were injected with the HRE-luciferase reporter plasmid via the tail vein using high-pressure injections. After incubation, mice were injected with PBS or LPS, or were subjected to a sham or CLP procedure. 6h later, livers were visualized using the IVIS SpectrumCT system. Log10 of the bioluminescent photon counts in liver of mice subjected to LPS or CLP and PBS and sham as control (n=3/group). All bars represent mean ± SEM. P-values were analyzed with two-way ANOVA. **P<0.01. [file Image_2.pdf]
